# Validity, Reliability, and Feasibility of Physical Literacy Assessments Designed for School Children: A Systematic Review

**DOI:** 10.1007/s40279-023-01867-4

**Published:** 2023-06-21

**Authors:** Lisa M. Barnett, Alethea Jerebine, Richard Keegan, Kimberley Watson-Mackie, Lauren Arundell, Nicola D. Ridgers, Jo Salmon, Dean Dudley

**Affiliations:** 1 Institute for Physical Activity and Nutrition, Deakin University, Melbourne, VIC Australia; 2 School of Health and Social Development, Deakin University, Melbourne, VIC Australia; 3 Centre for Sport, Exercise and Life Sciences, Coventry University, Coventry, UK; 4 Faculty of Health, University of Canberra, Research Institute for Sport and Exercise (UCRISE), Canberra, ACT Australia; 5 School of Exercise and Nutrition Sciences, Deakin University, Melbourne, VIC Australia; 6 Alliance for Research in Exercise, Nutrition and Activity, Allied Health and Human Performance, University of South Australia, Adelaide, SA Australia; 7 Macquarie School of Education, Macquarie University, Sydney, NSW Australia; 8 Institute for Physical Activity and Nutrition, School of Health and Social Development, Deakin University, Geelong, 3125 Australia

## Abstract

**Background:**

While the burgeoning researcher and practitioner interest in physical literacy has stimulated new assessment approaches, the optimal tool for assessment among school-aged children remains unclear.

**Objective:**

The purpose of this review was to: (i) identify assessment instruments designed to measure physical literacy in school-aged children; (ii) map instruments to a holistic construct of physical literacy (as specified by the Australian Physical Literacy Framework); (iii) document the validity and reliability for these instruments; and (iv) assess the feasibility of these instruments for use in school environments.

**Design:**

This systematic review (registered with PROSPERO on 21 August, 2022) was conducted in accordance with the Preferred Reporting Items for Systematic Review and Meta-Analysis (PRISMA) statement.

**Data Sources:**

Reviews of physical literacy assessments in the past 5 years (2017 +) were initially used to identify relevant assessments. Following that, a search (20 July, 2022) in six databases (CINAHL, ERIC, GlobalHealth, MEDLINE, PsycINFO, SPORTDiscus) was conducted for assessments that were missed/or published since publication of the reviews. Each step of screening involved evaluation from two authors, with any issues resolved through discussion with a third author. Nine instruments were identified from eight reviews. The database search identified 375 potential papers of which 67 full text papers were screened, resulting in 39 papers relevant to a physical literacy assessment.

**Inclusion and Exclusion Criteria:**

Instruments were classified against the Australian Physical Literacy Framework and needed to have assessed at least three of the Australian Physical Literacy Framework domains (i.e., psychological, social, cognitive, and/or physical).

**Analyses:**

Instruments were assessed for five aspects of validity (test content, response processes, internal structure, relations with other variables, and the consequences of testing). Feasibility in schools was documented according to time, space, equipment, training, and qualifications.

**Results:**

Assessments with more validity/reliability evidence, according to age, were as follows: for children, the Physical Literacy in Children Questionnaire (PL-C Quest) and Passport for Life (PFL). For older children and adolescents, the Canadian Assessment for Physical Literacy (CAPL version 2). For adolescents, the Adolescent Physical Literacy Questionnaire (APLQ) and Portuguese Physical Literacy Assessment Questionnaire (PPLA-Q). Survey-based instruments were appraised to be the most feasible to administer in schools.

**Conclusions:**

This review identified optimal physical literacy assessments for children and adolescents based on current validity and reliability data. Instrument validity for specific populations was a clear gap, particularly for children with disability. While survey-based instruments were deemed the most feasible for use in schools, a comprehensive assessment may arguably require objective measures for elements in the physical domain. If a physical literacy assessment in schools is to be performed by teachers, this may require linking physical literacy to the curriculum and developing teachers’ skills to develop and assess children’s physical literacy.

**Supplementary Information:**

The online version contains supplementary material available at 10.1007/s40279-023-01867-4.

## Key Points

**Table:** 

This review identified physical literacy assessments for children and adolescents based on a definition of physical literacy that incorporates physical, psychological, social, and cognitive domains.
Assessments with more validity/reliability evidence were the: Canadian Assessment for Physical Literacy version 2, Adolescent Physical Literacy Questionnaire, Passport for Life, Physical Literacy in Children Questionnaire, and Portuguese Physical Literacy Assessment Questionnaire.
Survey-based instruments were the most feasible to administer in schools.
Findings will be useful for researchers and practitioners who wish to assess children’s physical literacy in a school setting and need information on how instruments are classified in terms of current validity, reliability, and feasibility data.

## Introduction

There has been a surge of research interest in physical literacy in children and youth in the past 5 years (Web of Science: < 80 per year in 2014/15, 100 + in 2016/2017, 170 + in 2018/19, 250 + articles each year in 2020/21, and 800 + articles in 2022), which can partly be explained by the hypothesis that possessing greater physical literacy will enhance an individual’s likelihood of participating in lifelong physical activity [1]. Physical literacy has been defined in various ways [2–5] and for this paper, we have selected the Australian definition: “*Physical literacy is lifelong holistic learning acquired and applied in movement and physical activity contexts. It reflects ongoing changes integrating physical, psychological, social and cognitive capabilities. It is vital in helping us lead healthy and fulfilling lives through movement and physical activity. A physically literate person is able to draw on their integrated physical, psychological, social and cognitive capabilities to support health promoting and fulfilling movement and physical activity — relative to their situation and context — throughout the lifespan,*” as described in the Australian Physical Literacy Framework (APLF) [6, 7]. The APLF incorporates four domains (physical, psychological, cognitive, and social) and 30 elements of physical literacy within these domains that are based on the capabilities/capacities known to influence human movement [7].

This research interest is also reflected in publications and debate regarding how and whether to assess physical literacy [8–11]. This review follows a pragmatic perspective, maintaining that assessment is important to understand any individual, at any point, on their physical literacy journey and how they can best be supported. While there have been several reviews on physical literacy instruments [8–11], no review has comprehensively documented the validity and reliability of developed instruments for school-age children and youth. When selecting assessment instruments, it is important to be able to understand the degree of available validity evidence for the context, for example, the school setting. This enables an instrument to be selected based on its measurement properties. We can be more confident of our findings if the physical literacy measurements we use have stronger validity and reliability evidence. Another important aspect of the choice and use of instruments is their feasibility for collecting data in the given context [10, 12].

A recent scoping review identified that some of the latest approaches to defining and assessing physical literacy encompassed notions regarding physical, psychological, cognitive, and social learning [13]. While many instruments assess component parts of physical literacy [12, 14], for example, movement skills, our purpose was to capture instruments that have been purposefully designed to measure physical literacy as a holistic construct. The APLF is our benchmark of a holistic assessment model, as it incorporates four domains (physical, psychological, cognitive, and social) unlike many other instruments [8–11]. In addition, this work was commissioned by the Australian Sports Commission (the funders of the APLF) to identify and understand which instruments developed for use in school-aged children best mapped to the APLF. Thus, the purpose of this review was to: (i) identify instruments designed to measure physical literacy in school-aged children; (ii) map these instruments to the APLF; (iii) document the validity and reliability for these instruments; and (iv) assess the feasibility of use of these instruments in school contexts.

## Methods

### Initial Search of Reviews

Reviews (narrative and systematic) of physical literacy instruments in the past 5 years (2017 onwards) [located through Google Scholar using the terms ‘physical literacy’ and ‘review’ on 20 July, 2022] were used to identify instruments (subjective or objective) specifically designed for the purpose of assessing physical literacy in school-aged children in the school setting. In this review, including ‘physical literacy’ in the name of an instrument may not necessarily meet the review inclusion requirements. As the aim was to identify instruments designed to measure a holistic construct of physical literacy, instruments needed to assess at least three domains of the APLF (i.e., psychological, social, cognitive, and/or physical). Instruments that met these criteria and addressed additional elements outside of the APLF were also included.

### Search Terms and Databases

Searches were conducted by health faculty librarians on 20 July, 2022 for physical literacy instruments in school-aged children (not preschool or early years) that may have been missed/or published in (or since) the existing reviews in the past 5 years in six databases (CINAHL, ERIC, GlobalHealth, MEDLINE, PsycINFO, SPORTDiscus) [date range 1 June, 2017 to 30 June, 2022]. The search strategy, including all identified keywords and relevant subject headings (e.g., MeSH and Thesaurus terms), was adapted for each included information source. The key concepts and search terms were Concept 1: ‘Child’, Concept 2: ‘School’, and Concept 3: ‘Physical literacy’. Please see the Electronic Supplementary Material for the final search plan including alternative terms for the concepts. Table 1 reports the inclusion criteria for the review. Each screening step involved two authors with any issues resolved through discussion with a third author.

**Table 1:** Inclusion criteria for the screening process

**Stage 1 screening: abstract and title**	
*General criteria applicable to all papers*	
1. Language: published in English	
2. Article type: original research and reviews (narrative and/or systematic). Book chapters, case studies, and student dissertations (not conference abstracts)	
3. Sample: children (typically developing or not) with a reported mean age or age range between 5 and 18 years who are attending school	
4. Setting: school, e.g., primary, elementary, middle, secondary, and high (not early childhood)	
5. Topic: discusses physical literacy assessment	
*Implementation of assessment feasibility: specific criteria*	*Instrument reliability and/or validity: specific criteria*
Any study design	Any study design relevant to instrument development or validation
Uses words relevant to whether we can use (or not) this instrument/approach in a school setting, i.e., it mentions feasibility aspects (e.g., easy/hard to use and administer, time to complete, training of assessors, space needed to conduct, and equipment needed)	Has a purposeful approach to physical literacy assessment, i.e., the approach/instrument is explicitly designed to assess physical literacy (e.g., rather than standards developed for physical education)
Mentions teachers or schools and perspectives about physical literacy assessment (e.g., enablers such as links to curriculum, barriers such as time and school infrastructure)	About physical literacy instrument validity and/or reliability
	Must be an assessment that could be administered in a school setting (i.e., not measured through laboratory methods) within physical education or another lesson
	Also interested in articles that explore validity in terms of ‘*relations with other variables*’, in this case, the instrument measured against age, sex, another physical literacy instrument, or a motor skill instrument
**Stage 2 screening: full text**	
*Implementation of assessment feasibility: specific criteria*	*Instrument reliability and/or validity: specific criteria*
All above criteria in Stage 1 are met. No additional criteria	All above Stage 1 criteria are met
	Reports on a measurement method (qualitative or quantitative) relevant to assessment. If it is qualitative, the measurement approach must be specified, e.g., reference to a framework/model/approach/theory that relates to the physical literacy assessment method
	Instruments needed to assess at least three domains as listed in the Australian Physical Literacy Framework. If the instrument did not assess the physical domain, then the assessment still needed to be centred in the context of movement behavior. For instance, an assessment designed to mention social/cognitive/psychological elements during a non-sport/movement/physical activity context would be excluded. Sedentary behavior would be included if measured as part of the physical literacy assessment
	Reported information on measurement properties (quantitative assessments) or theoretical development (qualitative assessments)
	Instruments needed to be the most recent version of that instrument. *Note: this is only relevant to one instrument where the second version has been revised and improved*

### Instrument Synthesis

Instruments that met the included criteria were classified against the APLF (by one author and then checked with a second author) in terms of which of the 30 elements they assessed. Within the coding process, it was possible for two (or more) items in an instrument to be matched to only one element in the APLF. For example, *motivation* might be assessed by more than one survey item within an instrument/assessment. The converse could also apply if the item was assessed as meeting more than one of the APLF elements. For example, the item might measure psychological aspects of *engagement and enjoyment* and social aspects of *collaboration*. If instruments assessed additional elements to those assessed in the APLF, they were mapped to the appropriate domain or new domains were created.

### Instrument Validity and Reliability

The Standards for Educational and Psychological Testing [15] provided the theoretical framework for assessing validity and reliability. These standards espouse that rather than ‘validating an instrument’, validation is a process involving ongoing evidence about the property of test scores and the interpretations that stem from instrument use within a context. The Standards discuss validity in terms of five aspects: *test content* (from a literature review and content validity with experts)*, response processes* (face validity), *internal structure* (internal consistency, test–retest and/or inter-rater reliability, construct validity)*, relations with other variables*, and the *consequences of testing* (screening potential). Specifically, for *relations with other variables*, age, sex, motor skill competence, physical literacy, and physical literacy over time were considered and reported on. Physical activity was not included, as this was not always considered part of the definition of physical literacy. The included instruments were assessed for each of these validity aspects (by one author and then checked with a second author) and then the evidence categorized as: supporting (✓), partially supportive ( ~), not supported (x), or not yet tested/reported (–). Please see Table 2 regarding how this was operationalized for this review.

**Table 2:** Type of evidence according to the Standards for Educational and Psychological Testing, American Educational Research Association [1], and how this was applied in this review

Type of validity evidence	Explanation of evidence	Applied in this review
Content evidence	Whether the assessment content (scenarios, questions, response options, and instructions) reflects the intended construct. This might be based on prior instruments, expert review, and/or using a particular framework/model	Considered as partial evidence if only one aspect was performed (e.g., Delphi survey but not a literature review). The literature review did not have to be published separately, just evidence it was performed
Response process evidence	Refers to analyses that evaluate how well the rater’s (or responders’) responses align with the intended construct, including analysis of the thoughts or actions by responders/raters during the assessment	Needed to report evidence of responses for the intended population to be considered as supporting evidence
Internal structure evidence	Refers to data that evaluate the relationship among assessment items and how these relate to the overall construct of interest. This could be measures of reproducibility (reliability) but can also include analysis on items and factors (such as construct validity)	Considered as partial evidence if only one aspect was provided (e.g., an aspect of reliability but no evidence for construct validity)
Relationships with other variables evidence	About the reporting of statistical associations between assessment scores and other measures that have a specified theoretical relationship (includes concurrent validity). This type of validity can be termed criterion related and includes concurrent, predictive, convergent, and discriminate validity	For our purposes, this could include reporting the relationship between physical literacy and: age (would expect a positive association), sex (boys higher in motor skills), motor skills (where a physical literacy instrument has a motor skill component), physical literacy (as measured by another instrument) – (would expect a positive association) and over time
Consequences evidence	About the impact of the assessment itself and any decisions and actions that result (e.g., remediation following a below expected performance) and differences in scores among subgroups where performances ought to be similar	This could include factors that influence such decisions, such as development of a cut off score to indicate poor physical literacy (e.g., at what point can this be determined?)

### Feasibility

Feasibility within a school environment for each physical literacy assessment with more than *test content* evidence was assessed using a modified matrix developed previously [10]. Instruments with less validity evidence were not considered for feasibility, as an instrument arguably needs reliability and validity to be established first. This process documented feasibility according to cost efficiency (time, space, equipment, training, and qualifications required), but not acceptability in the way the previous framework conceptualized it (i.e., participant understanding, completed assessments [10]), as this is considered as *test content* evidence within the validity framework [15].

## Results

### Identification of Instruments from the Google Scholar Search of Prior Reviews

**Fig. 1:**
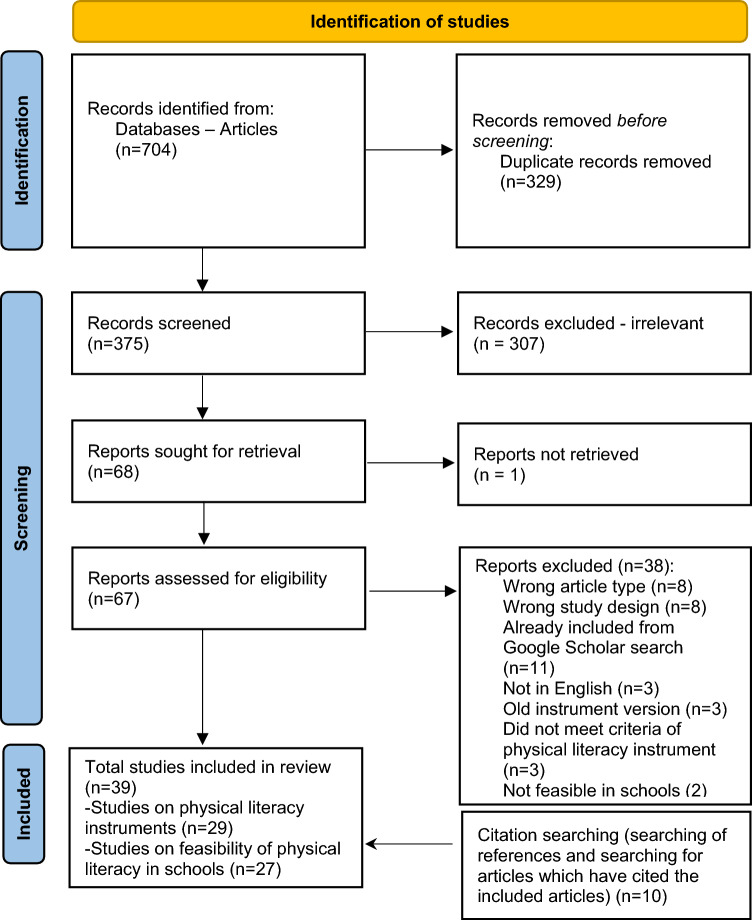
PRISMA Chart of identified studies for inclusion

Eight systematic or narrative reviews were identified (Fig. 1). These reviews included nine instruments (highlighted in underline and italics in this section) relevant for potential inclusion. Edwards et al. [8] used the global search term “physical literacy” to identify relevant instruments. Instruments did not meet our inclusion criteria if they typically focused on one domain of the APLF, particularly the physical (*n* = 22), the affective [also termed psychological] (*n* = 8), or the cognitive (*n* = 5) domains. The social domain was typically not assessed [8]. The Canadian Assessment for Physical Literacy CAPL (version 1) [16–18] was the only assessment that covered more than one domain of physical literacy but did not meet our inclusion criteria as it is not the most recent version of the CAPL.

Liu and Chen [19] undertook a narrative approach to physical literacy assessment that identified eight instruments. The Perceived Physical Literacy Inventory (PPLI) [20, 21], the Canadian Assessment for Physical Literacy version 2 (streamlined to 14 protocols rather than 25) [22–26], Passport for Life [27], and the Physical Literacy Assessment for Youth, specifically PLAY*fun*, PLAY*basic* (a shortened version of PLAY*fun*), PLAY*self*, and PLAY*coach* (counted as four instruments) [28] met our criteria*.* PLAY*parent* was not included as our focus was on assessments that could be performed in school. PLAY*coach* was seen as potentially relevant as a coach might be engaged in a sport program at school. Four did not meet our criteria, with two designed for the early years, one focused only on movement skills, and another was not explicitly designed to assess physical literacy [19].

Kaioglou and Venetasnou [9] conducted a review on physical literacy assessment instruments for use with children engaged in gymnastics and identified two approaches to physical literacy assessment; the first, to develop and use multi-component assessment instruments and the second, when existing standardized instruments were used. The first approach aligns with our inclusion criteria and the Canadian assessments already identified (PLAY tools, Passport for Life, and the Canadian Assessment of Physical Literacy) were the only instruments they identified that used this approach.

Shearer et al. [10] aimed to identify child assessments of physical literacy elements that were not necessarily branded as physical literacy assessments. Of the 52 potential assessment instruments identified, only the three named as physical literacy assessments were considered comprehensive by Shearer et al. [10] and met our inclusion criteria. These assessments (the Canadian Assessment for Physical Literacy, Passport for Life, and the Physical Literacy Assessment for Youth) have already been identified for inclusion in our review. Essiet et al. [14] also took a wide systematic approach beyond the physical activity and sport-related literature; however, the authors did not report any teacher proxy-report physical literacy instruments.

Jean de Dieu and Zhou [11] conducted a narrower systematic search and identified ten instruments, including four already identified in previous reviews [10, 19]. Two instruments mentioned in prior reviews did not meet our inclusion criteria. Additionally, Jean de Dieu and Zhou [11] identified the observed model of physical literacy [29] but this was not included in our synthesis, as it was still at the conceptual model stage. The instruments newly identified were the Chinese Assessment and Evaluation of Physical Literacy (CAEPL) [30] and the International Physical Literacy Association (IPLA) Physical Literacy Charting Tool (published 13 December, 2018, on the IPLA website https://www.physical-literacy.org.uk/library/charting-physical-literacy-journey-tool/). In the same year, Young et al. [31] published a review aiming to investigate physical literacy assessments in physical education, sport, or public health. The six identified assessment instruments were all identified in previous reviews for inclusion in our synthesis.

### Identification of Instruments from the Database Search

Through the database search, 39 papers relevant to the physical literacy assessment were identified. A total of 29 papers reported instrument reliability and/or validity. A total of 27 papers included information regarding the feasibility of assessment in schools [20 papers reported in Sect. 3.9 regarding the feasibility aspects captured in instruments and seven papers reported in the discussion on broader aspects of feasibility in schools (see Sect. 4)]. There was crossover between articles reporting validity and feasibility (Fig. 1).

Five additional assessments that were not included in the prior reviews met our inclusion criteria. These include the Adolescent Physical Literacy Questionnaire (APLQ) [32], the Physical Literacy in Children Questionnaire (PL-C Quest) designed for primary school-aged children [33, 34], the Physical Literacy self-Assessment Questionnaire (PLAQ) [35], and the Portuguese Physical Literacy Assessment Questionnaire (PPLA-Q) designed for adolescents in Grades 10–12 (aged 15–18 years) [36, 37]. One PhD thesis was also identified; Dong [38] developed the Perceptions of Physical Literacy for Middle-School Students (PPLMS).

### Instruments Included in Our Synthesis

A total of 14 instruments were included in our synthesis (nine from prior reviews and five from the updated search) [i.e., referred to by their acronyms that are listed alongside instrument details in Table 3. 1. APLQ, 2. CAEPL, 3. CAPL version 2, 4. IPLA, 5. PFL, 6. PLAQ, 7. PLAY*basic*, 8. PLAY*coach*, 9. PLAY*fun*, 10. PLAY*self,* 11. PL-C Quest, 12. PPLA-Q, 13. PPLI, and 14. PPLMS]. Six were from Canada, three from China, one from Australia, one each from Iran, Portugal, the UK, and the USA. There were seven self-report instruments, one designed for children (PL-C Quest), five designed for adolescents (APLQ, CAEPL, PPLA-Q, PPLI, and PPLMS), and one without an age specification (IPLA). One proxy-report instrument was designed for coaches (PLAY*coach*). A further four had mixed assessment approaches including self-report and observation (CAPL version 2, PFL, PLAY*fun & self*).

**Table 3:** Brief details of each instrument included in the review

Assessment instrument (alphabetical order of acronym)	Organization (if relevant)	Country	Intended target age of instrument	Assessment categories according to authors	Type of assessment	Targeted assessors (if not self-report)
1. Adolescent Physical Literacy Questionnaire (APLQ)	N/A	Iran	12–18 years	Psychological and behavioral Knowledge and awareness Physical competence and activity	Self-report	N/A
2. Chinese Assessment and Evaluation of Physical Literacy (CAEPL)	Shanghai University Sport	China	6–18 years	Intentions of physical activity Knowledge of physical activity Behaviors of physical activity Motor/sport skills for physical activity Physical fitness	Self-report	N/A
3. Canadian Assessment of Physical Literacy (CAPL version 2)	Healthy Active Living and Obesity Research Group (HALO)	Canada	8–12 years	Physical competence Daily behavior Motivation and confidence Knowledge and understanding	Self-report, objective	Trained assessors
4. Physical Literacy Charting Tool (IPLA)	International Physical Literacy Association	UK	No age specification	Motivation Confidence Physical competence Knowledge and understanding	Self-report	N/A
5. Passport for Life (PFL)	Physical & Health Education Canada	Canada	Children and adolescents	Active participation Living skills Fitness skills Movement skills	Self-report, objective	Trained assessors
6. Physical Literacy self-Assessment Questionnaire (PLAQ)	N/A	China	Grades 3–6 (8–12 years)	Physical competence Affective Knowledge and understanding Behavior of physical activity	Self-report	N/A
7. PLAY*basic*	Sport for Life Society	Canada	7–12 years	Physical competence	Objective	Trained assessors
8. PLAY*coach*	Sport for Life Society	Canada	7–12 years	Physical competence Knowledge and understanding	Proxy report	Coaches
9. PLAY*fun*	Sport for Life Society	Canada	7–12 years	Physical competence	Objective	Trained assessors
10. PLAY*self*	Sport for Life Society	Canada	7–12 years	Physical competence Knowledge and understanding	Self-report	N/A
11. Physical Literacy in Children Questionnaire (PL-C Quest)	Sport Australia	Australia	4–12 years	Physical Psychological Social Cognitive	Self-report	N/A
12. Portuguese Physical Literacy Assessment Questionnaire (PPLA-Q)	N/A	Portugal	15–18 years	Physical Psychological Social Cognitive	Self-report	N/A
13. Perceptions of Physical Literacy for Middle-School Students (PPLMS)	N/A	USA	11–13 years	Ability Confidence Desire	Self-report	N/A
14. Perceived Physical Literacy Inventory (PPLI)	N/A	China	12–18 years	Intentions of physical activity Knowledge of physical activity Behaviors of physical activity Motor/sport skills for physical activity Physical fitness	Self-report	N/A

*N/A* not applicable, *IPLA* International Physical Literacy Association

### Mapping Instruments Against the APLF

Table 4 shows the ALPF elements each instrument assessed; elements in italics are additional to those specified in the APLF. The instrument that assessed the most elements of the APLF was the PL-C Quest, which was designed to map to the APLF and therefore assessed the 30 APLF elements. The PFL (*n* = 20) and the PLAQ (*n* = 18) assessed the next highest number of elements, with both assessing all four domains of the APLF. The APLQ assessed 11 elements across four domains. The PPLI and the IPLA instruments assessed fewer elements (*n* = 8), but still across all four domains.

**Table 4:** Physical literacy assessments mapped against the Australian Physical Literacy Framework; additional elements in italics

Domain/element	1. APLQ﻿﻿	2. CAEPL	3. CAPLv2	4. IPLA	5. PFL	6. PLAQ	7. PLAY *basic*	8. PLAY*coach*	9. PLAY *fun*	10. PLAY *self*	11. PL-C Quest	12. PPLA-Q	13. PPLI	14. PPLMS	Element totals
Agility			✓					✓			✓			✓	4
Body composition		✓													1
Cardiovascular endurance	✓	✓	✓		✓	✓		✓		✓	✓		✓		9
Coordination			✓		✓	✓					✓				4
Flexibility	✓	✓				✓					✓				4
Movement skills	✓	✓	✓	✓	✓	✓	✓	✓	✓		✓			✓	11
Moving using equipment											✓				1
Muscular endurance	✓	✓	✓		✓						✓				5
Object manipulation		✓	✓		✓		✓	✓	✓		✓			✓	8
Reaction time											✓				1
Speed		✓				✓					✓				3
Stability/balance		✓	✓		✓		✓	✓	✓		✓			✓	8
Strength		✓				✓					✓				3
*Specific sport skills*	✓	✓													2
*Power*		✓													1
Physical sub-total	5	11	7	1	6	6	3	5	3	1	12	0	1	4	(65)
Confidence	✓		✓	✓	✓	✓		✓		✓	✓	✓	✓	✓	11
Connection to place											✓				1
Engagement and enjoyment	✓		✓		✓	✓				✓	✓	✓	✓	✓	9
Motivation	✓	✓	✓	✓	✓	✓		✓		✓	✓	✓		✓	11
Self-perception			✓	✓	✓	✓				✓	✓	✓	✓		8
Self-regulation (emotional)	✓				✓	✓				✓	✓	✓	✓	✓	8
Self-regulation (physical)					✓	✓					✓	✓			4
*Body image*					✓										1
Psychological sub-total	4	1	4	3	7	6	0	2	0	5	7	6	4	4	(53)
Collaboration				✓	✓	✓					✓	✓			5
Ethics					✓	✓					✓	✓			4
Relationships	✓			✓	✓						✓	✓	✓		6
Society and culture					✓						✓	✓	✓		4
Social sub-total	1	0	0	2	4	2	0	0	0	0	4	4	2	0	(19)
Content knowledge	✓	✓	✓		✓	✓		✓		✓	✓	✓	✓	✓	11
Perceptual awareness				✓				✓			✓				3
Reasoning					✓	✓					✓				3
Rules						✓					✓	✓			3
Safety and risk		✓				✓					✓				3
Strategy and planning				✓	✓						✓				3
Tactics											✓				3
Cognitive sub-total	1	2	1	2	3	4	0	2	0	1	7	2	1	1	(29)
Total elements	**11**	**14**	**12**	**8**	**20**	**18**	**3**	**9**	**3**	**7**	**30**	**12**	**8**	**9**	

*APLQ* Adolescent Physical Literacy Questionnaire, *CAEPL* Chinese Assessment and Evaluation of Physical Literacy, *CAPLv2* Canadian Assessment for Physical Literacy version 2, *IPLA* International Physical Literacy Association, Physical Literacy Charting Tool, *PFL* Passport for Life, *PLAQ* Physical Literacy Self-Assessment Questionnaire, (Physical Literacy Assessment for Youth,* PL-C Quest* Physical Literacy in Children Questionnaire, *PPLA-Q* Portuguese Physical Literacy Assessment Questionnaire, *PPLI *Perceived Physical Literacy Inventory, *PPLMS* Perceptions of Physical Literacy for Middle-School Students

The physical domain was the most assessed overall (*n* = 65), followed by the psychological (*n* = 53), cognitive (*n* = 29), and social (*n* = 19). The most assessed elements (defined as being in at least six of the 14 assessments) in the physical domain were *movement skills, cardiovascular endurance, *and then,* object manipulation*, and *stability/balance.* The most assessed psychological elements were *motivation* and *confidence*, *engagement and enjoyment*, and *self-regulation (emotional)* and *self-perception.* The most assessed cognitive element was *content knowledge*. The most assessed social element was *relationships*.

### Environmental Context

In eight assessments (IPLA, PFL, PLAY [all four] instruments, PL-C Quest, and the PPLI), the environmental context (e.g., land, snow, ice, water) was either specifically referred to or diversity in the environment was inherent in the items. The PPLI differed from the other instruments in that it did not refer to land or water as the environmental context, but specifically to ‘wild natural survival’.

### Additional Domains/Elements of Interest Identified to the APLF

Eight instruments measured this aspect. Some instruments had survey items covering a broad range of physical activity time periods and contexts. For instance, the APLQ asked about: hours of physical activity or exercise during the week and per day, and whether they did physical activity and exercise outside of school time or as a regular habit. The CAEPL included the domain of physical activity and exercise behavior in terms of: moderate- to vigorous-intensity physical activity, organizsed sports, active play, active transport, and experience in games/sports/events (within school/between schools/regional-national). The IPLA instrument included 15 survey items that investigated active participation (how often at school/home) in five movement domains: team sport (e.g., hockey, soccer), individual sport (e.g., golf, swimming), dance, gymnastics, and fitness activities (e.g., jogging, yoga). The PLAQ refers to: participation in sports activities (including sports classes and extra-curricular activities) and games no less than five times a week, and sports activities (including physical education classes and extracurricular activities) being not less than 1 h per day. The PPLMS asked about: frequency of aerobic exercises for at least 60 min per day and a minimum of five times per week, whether sports were played for at least 60 min per day, frequency of participation in a physical activity program, and participation in physical activities, for at least 60 min every day.

Two instruments had survey items that were more limited in the context. Passport for Life had items on the number of physical education classes per week, and time in physical activity each day and PLAY*coach* asked coaches about the physical activities and sports that an individual person participated in, but this information was not included in the overall score of the instrument items.

One instrument used a device-based assessment of physical activity. The CAPL version 2 used pedometers (steps each day over 7 days) and had an item asking the number of days with at least 60 min of moderate-to-vigorous physical activity. The CAPL also asked participants about the number of days in the past week that they were physically active for at least 60 min per day; recommended by their Delphi process [17].

Two of these eight assessments also included sedentary behavior. The CAEPL included screen-based time and homework time (this was included in their final model even though it did not reach expert agreement during content development of the instrument). The PLAQ had an item stating: “I spend more than 2 h on the electronic screen every day.”

In terms of the additional elements identified (beyond the APLF), in the physical domain, items referring to *specific sports skills* were included in two instruments (APLQ, CAEPL). *Body composition* was included in one instrument (CAEPL), while *power* and *body image* were each included in one instrument. Some elements that are part of the APLF were only assessed by the two instruments directly aligned to the APLF (*reaction time, connection to place*).

### Reliability and Validity Evidence for the Selected Instruments

A summary of validity and reliability evidence for each instrument is presented in Table 5. A narrative description of this evidence is presented below.

**Table 5:** Summary of validity and reliability evidence for each instrument

Instrument (alphabetical order)		Evidence for n validity aspects	1. Test content		2. Response processes	3. Internal structure			4. Relations with other variables					5. Consequences evidence
			Content validity (literature review/prior instruments)	Content validity (experts)	Face validity	Internal consistency	Test–retest and/or interrater reliability	Construct validity	Age	Sex	Motor skill	Physical literacy	Time	Screening potential
1. Adolescent Physical Literacy Questionnaire (APLQ)		3–4	✓	✓	✓	✓	✓	✓	~					**-**
2. Chinese Assessment and Evaluation of Physical Literacy (CAEPL)		1	~	✓	**-**	**-**	**-**	**-**	**-**	**-**	**-**	**-**	**-**	**-**
3. Canadian Assessment for Physical Literacy (CAPL) version 2	Canadian Agility and Movement Skill Assessment (CAMSA)	2–3	✓	✓	**-**	**-**	~	✓	✓	✓	**-**	**-**	**-**	**-**
	Motivation/confidence	2	✓	✓	**-**	✓	**-**	✓	x	**-**	**-**	**-**	**-**	**-**
	CAPL version 2	4	✓	✓	✓	~	**-**	✓	✓	✓	**-**	✓	**-**	**-**
4. IPLA Physical Literacy Charting Tool		1	~	✓	**-**	**-**	**-**	**-**	**-**	**-**	**-**	**-**	**-**	**-**
5. Passport for Life (PFL)		3–4	✓	✓	~	✓	✓	~	~	**-**	**-**	**-**	✓	**-**
6. Physical Literacy self-Assessment Questionnaire (PLAQ)		1–2	**-**	✓	**-**	**-**	**-**	✓	**-**	**-**	**-**	**-**	**-**	**-**
Physical Literacy Assessment for Youth instruments (PLAY)	*7.basic*	1–2	**-**	**-**	**-**	x	✓	**-**	✓	✓	✓	**-**	**-**	**-**
	*8.coach*	0	**-**	**-**	**-**	**-**	**-**	**-**	**-**	**-**	**-**	**-**	**-**	**-**
	*9.fun*	2	**-**	**-**	**-**	✓	✓	✓	✓	✓	✓	**-**	**-**	**-**
	*10.self*	1	**-**	**-**	**-**	✓	✓	-	**-**	x	**-**	**-**	**-**	**-**
11. Physical Literacy in Children Questionnaire (PL-C Quest)		3–4	✓	✓	✓	✓	✓	✓	**-**	~	**-**	**-**	**-**	**-**
12. Portuguese Physical Literacy Assessment Questionnaire (PPLA-Q)		3–4	✓	✓	✓	✓	✓	✓	**-**	~	**-**	**-**	**-**	**-**
13. Perceived Physical Literacy Inventory (PPLI)		2	**-**	**-**	**-**	**-**	**-**	✓	x	✓	**-**	**-**	**-**	**-**
14. Perceptions of Physical Literacy for Middle-School Students (PPLMS)		3–4	✓	✓	-	✓	**-**	✓	**-**	**-**	**-**	**-**	**-**	**-**

✓ met this aspect of validity evidence, - not reported, x reported but not considered to meet the criteria, i.e., poor value reported, ~ partially considered to meet criteria, *IPLA* International Physical Literacy Association

#### Instruments with Evidence of Test Content Only

Several instruments had evidence of *test content* only with one article located for each instrument. The CAEPL for school-aged children is in the conceptual stages of an assessment approach [30]. The IPLA instrument is available on the IPLA website (https://www.physical-literacy.org.uk/library/charting-physical-literacy-journey-tool/, accessed 14 July, 2022), and is developed from theoretical perspectives but no published validity or reliability data could be located. One paper that appears relevant to the IPLA approach highlighted considerations that organizations could make to develop methods to chart individuals’ progress [39].

#### Instruments with Evidence of Two Validity Aspects

The PLAQ (one article located [35]) used a grounded theory approach with students, parents, teachers, and experts to develop their physical literacy evaluation indicators for Chinese children in Grades 3–6, but they did not report a literature review; therefore, this was rated as partially meeting evidence for *test content* [35]. *Internal structure* was investigated using a factor analysis in a large sample (*n* = 1179) of Chinese children from randomly selected primary schools [35]. After an exploratory factor analysis, 16 items with low loadings were deleted and 44 items were retained. A confirmatory factor analysis then confirmed the structure (physical competence, affective, knowledge and understanding, physical activity) of the 44 reduced items [35].

Evidence for two validity aspects for the PPLI scale (three articles located [20, 21, 40]) in Hong Kong adolescents aged 11–19 years was reported [20]. Partial evidence for *internal structure* (a satisfactory three-factor structure but no information on reliability) and partial support for *relations with other variables* (male individuals had higher physical literacy levels than female individuals but perceptions of physical literacy were not impacted by age) was reported [20]. A translation into Turkish with 12-to-19-year-old adolescents investigated the PPLI (renamed as the Perceived Physical Literacy Scale for Adolescents [PPLSA]), reported further evidence of *internal structure* (a three-factor model structure with acceptable fit; internal consistency of 0.90 for whole scale; test–retest reliability ranged between 0.77 and 0.96) [40]. An earlier paper (2016) reported validity evidence of the PPLI in reference to teachers’ completion on behalf of themselves and thus this evidence was not considered as supportive of our population of interest (children) [21].

The PLAY instruments also have a range of publications with validity evidence (five articles reported in this section [28, 41–44] and one article mentioned in Sect. 3.8. [45]). There was mixed evidence, depending on the instrument, from mainly Canadian populations and one Croatian population [44]. Evidence for *test content* was not identified for any of the PLAY instruments. *Internal structure* of PLAY*fun* with 7- to 14-year-old individuals, with support for inter-rater agreement (ICC = 0.87) and a five-factor structure satisfactory model fit [41], was reported. There was also evidence for *relations with other variables* for sex and age (scores increased with age and in subscales such as object control boys were higher). PLAY*fun* and PLAY*basic* were investigated in children aged 8–14 years living in remote Canadian communities and further evidence of *internal structure for* PLAY*fun* (inter-rater reliability ICC = 0.78 and 0.82; α = 0.83–0.87) was provided [42]. *Internal structure* for PLAY*basic* was partially supported (inter-rater reliability ICC = 0.72 and 0.79; α = 0.56–0.65). *Relations with other variables* was reported again for age in terms of positive correlations [and PLAY*fun* (*r* = 0.23–0.39) and PLAY*basic* (*r* = 0.21–0.34)]. Additionally, both these PLAY instruments had large positive correlations with the Canadian Agility and Movement Skill Assessment (CAMSA) motor skill obstacle course (PLAY*fun r* = 0.47–0.60, PLAY*basic r* = 0.40–0.61) and small-to-moderate correlations with a self-reported measure of physical activity (PLAY*fun r* = 0.24–0.44, PLAY*basic r* = 0.20–0.42). A suite of PLAY instruments was tested in children aged 8–13 years [28]. Evidence of *internal structure* was supported for PLAY*fun* (internal consistency, α > 0.70; inter-rater reliability, ICC > 0.80) but only partially supported for PLAY*basic* (α = 0.47; inter-rater reliability ICC > 0.80). Test–retest reliability and factor validity were not assessed. There was also evidence of *relations with other variables* (male individuals scoring higher on PLAY*basic* and PLAY*fun* total scores; age positively correlated with PLAY*basic* and PLAY*fun* [*r* = 0.16–0.32]). PLAY*fun* and PLAY*basic* were also both positively correlated (*r* = 0.19–0.59) with another measure of motor competence (BOT-2).

Evidence of *internal structure* for PLAY*self* in children (aged 8–14 years) has been reported [43] with good reliability (α = 0.80, and test–retest reliability over 7 days, 0.87) and while the initial fit statistics were not ideal, when two items were removed the final fit statistics were satisfactory [43]. In the Croatian population of individuals aged 14–18 years, PLAY*self* had acceptable internal consistency for the components (the total score was not reported) and good test–retest reliability (0.85) [44]. Construct validity was confirmed according to the factor analysis of two significant factors; no other forms of construct validity were tested [44].

There was no evidence of *relations with other variables* (male individuals did not score differently to female individuals for the total PLAY*self* score) [43, 44]. No published validity evidence could be located for PLAY*coach* [28].

#### Instruments with Evidence of at Least Three Validity Aspects

There is an available body of evidence regarding validity evidence for the CAPL version 2 (11 articles in total, ten described in this section [18, 23, 25, 26, 44, 46–50], and one mentioned in Sect. 3.8. [45]). Evidence for *test content* has been published for Canadian children for: the movement skills assessment component (the CAMSA [18]), the domains of motivation and confidence [26], and the CAPL version 2 approach [23].

A Danish validation recently published evidence for *response process.* Elsborg et al. [46] selected the lowest grade levels (second grade) in Danish children on the basis they may have the most trouble to complete, and then conducted a pilot study of both the physical tests and the survey, followed by cognitive interviewing. As a result, the questionnaire administration was modified from paper to video-assisted (pictures and audio) for the children to complete unassisted on a tablet/computer.

Evidence regarding *internal structure* is supported overall, while internal consistency values show mixed evidence. The motivation and confidence domains are referred to in one paper [26], but these data could not be located in the additional files. However, the Danish study reported the motivation and confidence domains had good reliability (i.e., α = 0.90) [46]. A Chinese validity study also reported that motivation and confidence showed good internal consistency (α = 0.82), but the knowledge and understanding domain did not perform as well in that study (α = 0.52) [47]. The knowledge and understanding domain was assessed for test–retest reliability in a Croatian population of 14- to 18-year-old individuals with mixed results at the item level (total score not reported) [44].

Test–retest reliability for the CAMSA can be considered as partially supported, with excellent values reported for the completion time (ICC = 0.99) but lower values for the skill score reliability (ICC = 0.46 over a 2- to 4-day test interval and ICC = 0.74 over a longer interval) [18]. Published test–retest reliability for other aspects of the CAPL version 2 was not identified.

Evidence for the factorial structure of the domains of motivation and confidence [26], the factor structure of CAPL scores, and the contribution of each domain to the overall physical literacy score has been reported [25]. Subsequent Danish [46] and Chinese studies have reported acceptable model fits and factor loadings [47].

*Relations with other variables* for the CAPL version 2 is also generally supported. The CAMSA has reported convergent validity regarding motor skills in Canadian (i.e., age increasing and male sex) [18], Greek [49, 50], and Chinese children [47]. The Danish study also examined *relations with other variables*, with the CAPL version 2 score explaining 31.4% of the variance in physical education teacher ratings [46]. The CAPL was also modified (new protocols for the CAMSA and knowledge and understanding) for use with adolescents (aged 12–16 years) in Grades 7–9 (CAPL 789), with evidence of *relations with other variables* (i.e., physical competence increased with age and boys performed better on the CAMSA) [48]. In the Croatian sample, the knowledge and understanding domain did not show a difference according to sex [44].

Three articles regarding validity were located for the PPLA-Q [36, 37, 51]. Note that one article appears as a pre-reviewed version [36]. *Content evidence* (literature review, an analysis of the APLF, and expert validation) for the PPLA-Q for adolescents in Grades 10–12 (age 15–18 years) and *response process* evidence (gathered from interviews with students in the target age group) has been reported [37]. *Internal structure* was only partially evident in this paper (internal consistency > 0.70 in 10 of 16 scales, although problematic items were modified and tested with further cognitive interviews). In a subsequent paper that aimed to investigate the cognitive module of the PPLA-Q, more evidence concerning *internal structure* was provided (final model fit the data); however, the test–retest reliability was classified as poor to moderate (data not shown) [51]. Another paper aiming to test construct validity of the psychological and social modules of the PPLA-Q reported evidence of internal structure (as assessed though item dimensionality and convergent and discriminant validity and reliability, i.e., internal consistency > 0.80; test–retest reliability values between 0.66 and 0.92 across the eight scales) [36]. Therefore, the PPLA-Q was considered to meet the criteria for *internal structure* overall. Evidence of *relations with other variables* was partially supported for sex, with evidence of differential item and test functioning across sex groups reported in one item but with no significant effect at the test level [36].

The PL-C Quest (two articles located [33, 34]) has evidence of *test content* (literature review, experts) and *response processes* (interviews with children) in Australian school children aged 5–12 years [34]. A subsequent paper provided evidence in Australian children aged 7–12 years for *internal structure* (internal consistency, α = 0.92; test–retest reliability over 16 days, ICC = 0.83; satisfactory fit for a Confirmatory Factor Analysis model with four domains and a higher order factor of physical literacy) [33]. *Relations with other variables* was partially supported as boys reported higher values in some of the items relating to the physical domain, but not for the movement skill items.

Validity evidence (from one article [32]) for the APLQ in a large sample of Iranian adolescents aged 12–18 years was reported [32]. *Test content* (literature review, experts), *response process* (adolescent opinion), *internal structure* (internal consistency α = 0.95; test–retest reliability over 11 days, ICC = 0.99; construct validity confirmed three factors: psychological and behavioral, knowledge and awareness, and physical competence and physical activity) were all supported. There was some evidence for *relations with other variables* (correlated with the PPLI, *r* = 0.79 for the total score).

Three articles were located for the PFL, two in this section [27, 52] and one described in Sect. 3.8. [45]). Lodewyk and Mandigo [27] published *test content* evidence (consultative process and expert feedback) for PFL in Canadian children and adolescents (Grades 4–9, age not reported). Data from a pilot test of a draft of the Grade 10–12 PFL (sample of 642 students) were part of the development process. Feedback resulted in minor modifications to the wording of some items [52].

Some evidence of *response processes* was also reported. While more than 90% of teachers reported Grade 7–9 students were able to understand the assessments, this percentage was lower for Grade 4 and 5 students (living skills: 71%; active participation: 66%) [27]. The teachers said the year 10–12 students could follow and understand the active participation and living skills items [52].

There was support for the *internal structure for* the younger students in terms of reliability [internal consistency (> 0.60); inter-rater agreement (0.65–0.82); test–retest reliability (*r* = 0.72–0.89)] and initial partial support for construct validity (each item within each scale had strong factor loadings [0.53–0.81] and scale correlations within each PFL component had positive significant associations) [27]. For students in grades 10–12, there was also support for reliability [internal consistency (α > 0.83)]. Further, each item (bar two that were later omitted) had at least a satisfactory factor loading (0.30–0.81), and the extracted factor explained a satisfactory proportion of variance [52]. Finally, there was some evidence for *relations with other variables,* as authors reported predictive consistency between scales and components over the testing period of 2 years for the different year groups [52].

For the PPLMS (one PhD thesis located [38]), evidence of *content validity* was based on a construct map and literature review All scale items were aligned with the National Standards and grade level outcomes for K-12 PE published by SHAPE and theories of physical literacy prescribed by Whitehead Expert feedback was provided by academic staff [38]. There was evidence of *internal structure*. There was good internal consistency reliability for each subscale and the total 22-item instrument (0.93) and adequate construct validity (an exploratory factor analysis found a 22-item instrument with four subscales and a subsequent confirmatory factor analysis confirmed the first model [χ2/df = 1.487, root mean square error of approximation = 0.067, standardized root mean square residual = 0.062, Tucker Lewis Index = 0.903, Comparative Fit Index = 0.914]). All items loaded greater than 0.40 in the final model [38].

### Gaps in Evidence

Only one study published *consequences evidence* [45]. That study evaluated the sensitivity and specificity of 40 screening tasks (including the PFL and PLAY motor skills, older version from 2013) to determine which tasks could identify children in need of support. The CAPL (version 1) reported children with a low or high body mass index z-score and children with a predilection score towards physical activity less than 31.5/36 points were the most likely to have a CAPL physical literacy score below the 30th percentile [45]. While two of the instruments in this paper were not current versions, these findings are reported here as it was the only evidence located related to this validity aspect*.* No study reported on using any of the included instruments in children with disability.

### Feasibility of the Physical Literacy Assessment Instruments

Only the instruments with more than one aspect of validity evidence were considered for feasibility. (i.e., 1. APLQ, 3. CAPL version 2, 5. PFL, 6. PLAQ, 7. PLAY*basic*, 8. PLAY*coach*, 9. PLAY*fun*, 10. PLAY*self,* 11. PL-C Quest, 12. PPLA-Q, 13. PPLI, and 14. PPLMS). Please see Table 6 for information on feasibility. The physical literacy assessment instruments need to be considered separately in terms of their approach. The instruments with mixed assessment approaches that include observation require more time to administer.

**Table 6:** Summary of feasibility aspects captured in the instruments (20 articles located)

Instrument (alphabetical order)		Time	Space	Equipment	Assessors/training	Availability	Data capture	Other comments
1. Adolescent Physical Literacy Questionnaire (APLQ﻿﻿)		“easy to use and not a long time” [32]	Seated survey	N/A	NR	Available [32]	Pen and paper	
2. Canadian Assessment for Physical Literacy (CAPL) version 2	The Canadian Agility and Movement Skill Assessment (CAMSA)	Median completion = 17 s for each repetition of assessment. 25 min for 20 children (examiner demonstration = 1 min; 17 s/practice trial × 2 trials/child × 20 children = 12 min; 17 s/measured trial × 2 trials/child × 20 children = 12 min) [18]	Travel a total distance of 20 m while completing 7 movement skill tasks	Hoops, soft ball, wall target, soccer ball, 2 cones	Examiners had extensive experience in movement skill analysis. Graduate degrees in kinesiology and up to 5 h of additional training specific to protocol [18]	Freely available CAPL-2 website (www.capl-eclp.ca)	Two examiners required to administer and score the assessment	Program available for data analysis [54]
	CAPL version 2 (including CAMSA)	Maximum completion time about 30 min per child with two assessors [49] “.. organised in groups of 25–30 children, usually conducted by three appraisers in a single session of 90ʹ or alternatively, in two sessions across two consecutive days.” [50] “.. two appraisers (one male and one female) for separately evaluating the aerobic test, motor skills and muscular endurance test” [47]	Combination of seated and activity (as per the CAMSA) “Due to limited space, all participants ran between two markers set 15 m apart,” [47]	As above per CAMSA	Specialists in PE [49] PE specialists and sport professionals [50] “18 h training workshop included a theoretical course and two practical courses, with an examination” [47]	Training materials available on website Freely available CAPL-2 website (www.capl-eclp.ca)	Pen and paper and objective measures	“Additional investigations to evaluate the burden of CAPL-2 for examiners and participants are recommended” [23] “To help the children who had difficulties reading, a video where all items of the questionnaires were read out loud, while the text appeared on the screen, were made.” [46]
	CAPL 789 (modified for children in Grades 10–12)	“The CAPL 789 can be completed in approximately 60 min per student. A group of 25 children can be assessed by a team of 5 evaluators in approximatively 90 min” “The addition of the throw/catch task also extended the completion time for the CAMSA component” [48]	As per the original CAMSA with the addition of a wall for the throw and catch additional task	As above per CAMSA and a tennis ball	“Postsecondary or graduate degrees in physical activity science (e.g. kinesiology, exercise physiology) and were appropriately trained” [48] CAPL Training Manual (https://www.capl-ecsfp.ca/wpcontent/uploads/capl-manual-english.pdf) and CAPL Training Videos (https://www.capl-ecsfp.ca/capl-trainingvideos/)	Contact author	Pen and paper and objective measures	“Despite the development of a standardised protocol and the confirmation of participants that they understood the different assessments, it is clear that it is difficult to determine if this really was the case with such a long protocol” [48]
5. Passport for Life (PFL)		Between 2.5 and 6 classes [27] Class time needed to set up class accounts and for students to complete online profile [53]	Four stations circuit needs an area the size of a badminton court	Ball, soccer ball, cones	Several teachers expressed need for video aids to help with movement and fitness skills and administration. In response, video tutorials and demonstrations were added to website [27] Online videos outlining processes to register students and use assessment instruments are available [53]	Freely available (https://passportforlife.ca/)	Online database [27] “Teachers can record the students’ assessment results immediately on an iPad/ computer or via a printable handout. If they choose the printable handout, they will have to transfer the results to each student’s individual account.”	“For each fitness item, teachers simultaneously assessed as many students as they deemed feasible” [27] “.. most prevalent administrative concern was amount of class time spent completing PFL with 43% reporting that it took an unreasonable amount of time” [27] “teacher resources were useful, relevant, and easy to use and access, easy to register students and enter and interpret data and results in online system” [52] “Teachers’ lowest ratings (45%) were that PFL assessments—especially those for movement and fitness skills—took a reasonable amount of time to complete (on average 4–6 classes) “ [52]
6. PLAQ		NR	Seated survey	N/A	NR	Available in paper [35]	Pen and paper	
Physical Literacy Assessment for Youth instruments (PLAY)	*7. Basic*	< 5 min [28]	Skills assessment	Pylons (cones) Large wall, tennis ball, soccer ball	Based on experienced assessor [28]	Freely available https://play.physicalliteracy.ca/play-tools/playbasic	Pen and paper	“If the teacher was looking for a physical literacy score and administered the entire test, it would be extremely time consuming” [53] Online assessment requires students to be enrolled electronically in system [53]
	*8. Coach*	NR	Seated survey	N/A	NR	https://play.physicalliteracy.ca/play-tools/playcoach	Pen and paper	
	*9. Fun*	10 min [41]	Skills assessment	Pylons (cones) Large wall, tennis ball, baseball tee, baseball bat, basketball, soccer ball	“Before testing, all assessors completed more than 10 h of training, including an orientation session led by the designer of the measure, Dr. Dean Kriellaars” [41]	Freely available https://play.physicalliteracy.ca/play-tools/playfun	Pen and paper	
	*10. Self*	5–10 min [43]	Seated survey	N/A	Minimal training -administered by a teacher trained in administration of the PLAY*self* tool [43]	Freely available https://play.physicalliteracy.ca/play-tools/playself	Pen and paper, later entered onto an online database [43]	
11. Physical Literacy in Children Questionnaire (PL-C Quest)		Most children < 20 min; median of 11.5 min [33]	Seated survey	N/A	User guide for administrators available at Australian Sports Commission https://www.sportaus.gov.au/physical_literacy/resources	Request to the Australian Sports Commission	Pen and paper; also, online version	
12. Portuguese Physical Literacy Assessment Questionnaire (PPLA-Q)		Average time 27 min [37]	Seated survey	N/A	N/R	In supplementary materials [37]	Pen and paper	
13. Perceived Physical Literacy Inventory (PPLI)		8–10 min average [20]	Seated survey	N/A	NR	Available [20]	Pen and paper	
14. Perceptions of Physical Literacy for Middle-School Students (PPLMS)		“expected it would take 20 min to respond to all items [38]	Seated survey	N/A	NR	Contact developer [38]	Pen and paper	

*N/A* not applicable, *NR* not reported

Considering just the assessment approaches that use a survey only, the shortest was 8–10 min to complete/administer (PPLI), followed by the PL-C Quest (median 11.5 min), and then the PPLA-Q (27 min). The remainder did not report a completion time (APLQ, PPLI, PLAQ, PPLMS).

The PLAY instruments seem to take the least time with the objective components (PLAY*fun* or *basic*) taking 5–10 min, the seated component (PLAY*self*) also taking 5–10 min to administer and PLAY*coach* does not have an administrative time reported. However, one study noted that the PLAY tools were time consuming as a whole package [53]. The motor skill component of the CAMSA can be completed quickly by a whole class group rather than one-on-one (25 min for 20 children), but it is not clear how long the entire CAPL version 2 takes to complete. One study described the time required to complete CAPL-2 as burdensome [23].

A recent paper documents an R analysis package [54] that automates the results process (capl R package [open source], to compute and visualize scores and interpretations from raw data). This could potentially assist in feasibility for researchers, but likely not for the feasibility of administration in school settings by teachers as this would require specialist knowledge to run the package. The whole PFL assessment is reported to take between two and six lessons to complete for a class group of children, with this being reported as an unreasonable amount of time [27]. These instruments (CAMSA, PFL, Play*basic*, and PLAY*fun)* also require space, equipment for the objective components, and a level of training for administering these sections. The CAMSA requires two staff to administer and while the number of staff is not reported for PLAY*fun/basic* and the PFL, it is likely that two staff would also be needed for a class, i.e., one to administer and one to supervise the remaining children.

## Discussion

This review identified 14 tools, mainly from Canada, designed to measure physical literacy in children and adolescents. Overall, the assessment approaches with more validity evidence (at least three to four validity aspects according to the standards developed by the American Educational Research Association [15]) were the PL-C Quest and PFL for children, the CAPL version 2 for older children/younger adolescents and the APLQ and PPLA-Q for adolescents. Note that for the PPLA-Q, one supporting article did not appear to be peer reviewed [36]. Additionally, whilst the PPLMS did have three to four aspects of validity for middle school-aged children, data were also not peer reviewed [38].

Even though these assessments had more validity and reliability evidence than other assessments, there was little evidence for consequences evidence. A recent paper has begun to question the consequential validity of physical literacy assessment instrument use (specifically CAPL version 2) in physical education settings [55]. It is also questionable whether determining a ‘cut-off’ for poor physical literacy is a useful approach for a strengths-based approach to physical literacy. There was also a lack of evidence regarding the ability of instruments to be sensitive to change. This is an important aspect for consideration when using instruments to measure change after an intervention. It is important to also note that seeking validity evidence is a journey, and thus some instruments developed more recently have not had the same time frame to develop validity evidence.

A clear gap for all assessments is validity and reliability evidence for instruments when the population includes children with disability. For example, one of the studies on the PFL noted that a gap was understanding students with special needs [52]. Instruments such as the PL-C Quest may offer opportunities here for children with intellectual disability because of the pictorial nature. There is emerging evidence of its utility for this population from a dissertation where it was used with adults with intellectual disability [56]. Although considering the diversity of disability experienced in children, adaptations of physical literacy assessment instruments may need to be tailored to individual disability populations, and this is an area that warrants further investigation.

When considering instrument breadth in terms of domain, the PL-C Quest was designed to map to the APLF and therefore assessed four domains (and 30 elements) of physical literacy. Other instruments that assessed more than ten elements across all four APLF domains were the PFL, PLAQ, PPLA-Q, CAEPL, CAPL version 2, and APLQ. Some instruments added additional domains and/or elements to those included in the APLF, potentially adding to a holistic mapping of physical literacy. Eight assessments incorporated physical activity (including sedentary behavior for two instruments) as an additional domain to the APLF. The position of the expert panel during the initial development of the APLF was that physical activity can be considered a consequence *and/or* antecedent of physical literacy, but not as an essential domain of physical literacy [57]. What this means in practical terms is that an individual may have high levels of physical literacy but not be active at that present time because of an injury or other personal circumstances, and thus the activity level is not always a reflection of an individual’s physical literacy.

Another aspect of instrument breadth or holism is the range of elements assessed. An additional element in the physical domain (*specific sports skills*) was added to three instruments (APLQ, CAEPL, PPLMS), with these instruments designed for adolescent populations. The addition of sports may make the instruments more relevant to adolescents, as the context of skill performance is then acknowledged. This supports the psychological theory that as children cognitively develop, their capacity to self-report in the physical domain changes to one that is more differentiated [58]. Other additional elements to the APLF were quite rare, i.e., *power* and *body image* were each added to one instrument and *body composition* was added to one instrument. *Power* could be a relevant addition to a holistic framework, although this would increase the number of physical elements and this domain already outweighs the other domains. *Body image* may be an important psychological element to consider including in a holistic physical literacy framework, as a scoping review identified positive body image as linked to physical activity and sport behaviors in adolescents (30% of the study samples) [59]. Including body composition as an element is like including physical activity behavior as a domain, in that can be perceived as reflecting a potential outcome and/or precipitator of physical literacy rather than necessarily being an indicator of physical literacy.

Survey-based instruments are the most feasible to administer in school settings and they can potentially reach larger populations/samples as a result, with the shortest being the PPLI and PL-C Quest. However, a key reason these instruments are shorter is that they do not provide an objective assessment of movement skills or fitness and therefore do not require more than one teacher to administer. Some instruments included an objective assessment of motor skill (CAPL version 2 and PLAY instruments, PFL), with the CAMSA (part of the CAPL version 2) reasonably efficient to administer as it is done as a class group (although two teachers are needed). Motor skill competence is an important component of physical literacy [1], and objective assessment is very well developed in the motor competence field with a plethora of reliable and valid assessment approaches to choose from [60–62]. Similarly, an objective assessment of cardiorespiratory and muscular fitness could be considered important to include. When using motor skill assessments as part of a physical literacy assessment, it is worth considering using a strength-based approach as opposed to deficiency testing.

A broader consideration of feasibility (seven articles located) is whether school personnel have the capacity, interest, and requirement to implement a physical literacy assessment. This discussion goes beyond the choosing of assessments for the school setting [12]. The need for teachers’ assessment of physical literacy in schools has been advocated whilst recognizing that Australian teachers had varying levels of understanding of the concept [63]. Two other Australian studies reported that health and physical education teachers’ understanding and operationalization of physical literacy in practice is limited, despite them largely being supportive of physical literacy [64, 65]. One of these studies recommended greater investment in studies that demonstrate how physical literacy supports the objectives of health and physical education [64].

Not having an explicit link to the curriculum is likely to be a primary barrier to physical literacy assessment in schools [65]. The instruments we have reviewed may have been originally designed to meet the needs of a particular curriculum. However, if such information was not explicitly reported in the articles identified in our search, then it was not reported. This problem is compounded when teachers’ personal physical capabilities are underdeveloped, as reported in a study of 57 pre-service teachers [66]. These authors contend greater attention to practical and physical learning experiences is required to develop teaching competencies [66]. A potential solution is physical literacy introduced as an additional proposition in the curricula (joining educative outcomes, strengths-based approach, health literacy, critical inquiry, and valuing movement) [67]. However, this contrasts with those who argue for the introduction of physical literacy as a general capability in the health and physical education curriculum, highlighting the ongoing discussion and divergence around the enactment of physical literacy in schools [68].

The strengths of this review include a thorough search, a comprehensive approach to validity assessment, and broad coverage of feasibility. Applying instruments to the APLF may be seen as a limitation depending on what definition of physical literacy the reader subscribes to, but even so, for those interested in physical literacy assessments that span multiple domains, this process should still have value. It also provides a template approach for others wishing to follow a similar process with other frameworks. It is important for transparency to anchor any physical literacy paper within the definition subscribed to. For example, an earlier paper conducted a conceptual critique of three Canadian physical literacy assessment instruments for school-aged children in terms of how well they related to Whitehead’s conception of physical literacy [53]. Reporting the theoretical standpoint and definition of physical literacy has also been recommended for the reporting of physical literacy interventions [69]. Even though our focus for this review was school-aged children, physical literacy is a lifespan concept and documenting the validity and reliability of instruments to assess physical literacy in the early years of children and adults are also worthy future endeavors.

## Conclusions

A total of 14 physical literacy assessment instruments were identified, with at least five (APLQ, PFL, PL-C Quest, PPLA-Q, and PPLMS) having evidence for at least three validity aspects. Three instruments assessed four domains of the APLF and more than half the elements (the PL-C Quest, PFL, and the PLAQ). Survey-based instruments were the most feasible to administer in schools, although a comprehensive assessment may arguably include some objective assessments.

## Supplementary Information

Below is the link to the electronic supplementary material.

Supplementary file1 (DOCX 65 KB)

## Declarations

### Availability of Data and Material

All data generated or analyzed during this study are included in this published article (and its supplementary information files.
