# Supplementary material for: Validity, Reliability, and Feasibility of Physical Literacy Assessments Designed for School Children: A Systematic Review
Source: Sports Med. 2023 Jun 21;53(10):1905–29. doi: 10.1007/s40279-023-01867-4 (PMC10504218; doi:10.1007/s40279-023-01867-4)
Supplement: Supplementary file 1 — Supplementary file1 (DOCX 65 KB) [file 40279_2023_1867_MOESM1_ESM.docx]

# Supplementary File 1: Search strategy

The search strategy was developed with the assistance of a university health librarian with expertise in advanced database searching and aimed to locate published literature only. A systematic search of six bibliographic databases (CINAHL, ERIC, Global Health, MEDLINE Complete, APA PsycInfo and SPORTDiscus with Full-text) was undertaken to identify articles on the topic. The search strategy, including all identified keywords and relevant subject headings (e.g., MeSH and Thesaurus terms), was adapted for each included information source. Search terms and subject headings are detailed in the tables below and an example search is given for ERIC via the EBSCOhost database.

The final search was completed on: 20 July 2022.

Restrictions applied:

- Language: English language only
- Publication or release period: 01 June 2017 – 30 June 2022

**Key concepts and search terms**

|  | **Term** | **Alternative terms** |
| --- | --- | --- |
| **Concept 1** | Child | Student, pupil, school-age, youth, (pre)adolescent, teen, boy, girl, young person |
| **Concept 2** | School |  |
| **Concept 3** | Physical literacy |  |

**Subject headings**

|  | **CINAHL**  **(MeSH)** | **ERIC**  **(Thesaurus)** | **Global Health**  **(Thesaurus)** | **Medline (MeSH)** | **Psych Info**  **(Thesaurus)** | **Sport-discus**  **(Thesaurus)** |
| --- | --- | --- | --- | --- | --- | --- |
| **Concept 1:**  **Child** | Child; Adolescence | Children; Youth; adolescents; young children; preadolescents | Children; Adolescents; Youth; children | Child; Adolescent |  | School children; children; teenagers; youth |
| **Concept 2:**  **School** | Schools; Schools, secondary  Schools; middle, Schools; elementary | Schools; Elementary schools,  Middle schools, Secondary schools | Schools; Elementary schools;  High schools  Primary education | Schools | Schools; Elementary schools; Middle schools; Junior high schools;  High schools | Schools |
| **Concept 3:**  **Physical literacy** |  |  |  |  |  |  |

**Example electronic Database search: ERIC via EBSCOhost**

| **Concepts** | **#** | **Searches** |
| --- | --- | --- |
| Concept 1 – field labels | S1 | TI child* OR AB child* |
|  | S2 | TI youth OR AB youth |
|  | S3 | TI teen* OR AB teen* |
|  | S4 | TI adolescen* OR AB adolescen* |
|  | S5 | TI “school* age*” OR AB “school* age*” |
|  | S6 | TI boy* OR AB boy* |
|  | S7 | TI girl* OR AB girl* |
|  | S8 | TI student* OR AB student* |
|  | S9 | TI pupil* OR AB pupil* |
|  | S10 | TI Preadolescent* OR AB Preadolescent* |
|  | S11 | TI Pre-adolescent* OR AB Pre-adolescent* |
|  | S12 | TI “young* people*” OR AB “young* people*” |
|  | S13 | TI “young* person*” OR AB “young* person*” |
| Concept 1 – Subject labels (Thesaurus) | S14 | DE "Children" |
|  | S15 | DE "Preadolescents" |
|  | S16 | DE "Young Children" |
|  | S17 | DE "Adolescents" |
|  | S18 | DE "Youth" |
| Combine terms with ‘OR’ | S19 | S1 OR S2 OR S3 OR S4 OR S5 OR S6 OR S7 OR S8 OR S9 OR S10 OR S11 OR S12 OR S13 OR S14 OR S15 OR S16 OR S17 OR S18 |
| Concept 2 – field labels | S20 | TI school* OR AB school* |
| Concept 2 – Subject labels (Thesaurus) | S21 | DE "Schools" OR DE "Elementary Schools" OR DE "Middle Schools" OR DE "Secondary Schools" |
| Combine terms with ‘OR’ | S22 | S19 OR S20 OR S21 |
| Concept 3 – field labels | S23 | TI “physical* litera*” OR AB “physical* litera*” |
| Combine terms with ‘AND’ | S24 | S22 AND S23 |
